# Ageism, menopause, and health disparities in Latin America

**DOI:** 10.1016/j.lana.2023.100638

**Published:** 2023-11-20

**Authors:** The Lancet Regional Health – Americas

Hot flushes, night sweats, low mood, anxiety, poor cognitive performance, reduced sex drive, and vaginal dryness are some examples of symptoms of menopause. Clinically, menopause refers to the last menstruation and post-menopausal phase is the period of transition from reproductive to non-reproductive phase. Although not a disease, these symptoms can affect all women and people who menstruate after certain age, usually between 45 and 55 years old, and the post-menopausal phase can last up to three decades. Despite being a natural biological process of the human body, the menopause transition can affect disease risk and is associated with physiological, behavioural, and psychosocial changes. Women from Latin America are no exception when it comes to stigmatisation of their symptoms, suffering from depression and adverse health behaviours that can drive them not to seek help or treatment to alleviate their symptoms. Most don’t even talk about menopause and many who experience it suffer quietly.

There has been debate as to whether the use of hormonal replacement to treat menopause symptoms is the best option. For example, hormonal therapy is still the most effective treatment for menopausal symptoms to prevent bone loss or cardiometabolic disease risk. But there are many components in women’s health that need to be accounted for when they start the therapy. Recently The Latin America and the Caribbean Code Against Cancer, launched by IARC, PAHO, and WHO groups, recommended that people refrain from using hormonal therapy due to its relation with breast cancer, unless the physician recommends it. Lifestyle can be a potential solution for menopausal symptoms. A recent study showed that menopausal status and key metabolic health indicators, such as visceral health, inflammatory, and glycaemic postprandial responses, are partly mediated by diet and gut bacteria abundance, and that lifestyle can affect menopausal symptoms. However, this cohort focused on a population from the UK, and Latin American populations have different lifestyles.

Populations in Latin America are ageing and by 2030, the population of people older than 60 years will exceed that of people younger than 5 years. Brazil, a country comprised of mostly women according to the 2022 census, has a higher ageing index compared with previous years. An individualised approach that targets the benefits and risks of the therapeutical approaches is needed, with personalised counselling to promote a healthy lifestyle. The recommendations by the Code Against Cancer might be difficult to adhere to, especially when clinicians are not fully aware of the available treatments on menopausal symptoms, as pointed out by a qualitative study that focused on the understanding of health on women nurses from Minas Gerais, Brazil. Finally, those experiencing menopause can be directly affected by the paucity of support and knowledge from health-care personnel.

The mental health of those who experience menopause is affected not only by hormonal changes, but also by the society, as women fear negative attitudes towards them and suffer from gendered ageism, belittlement, and misogyny. Quality of life is affected by symptoms, disrupting work and personal life, and women can feel embarrassed by some of the symptoms they face. Although few studies focus on the menopausal health of Latin American women, some reported that they have higher prevalence of vasomotor symptoms, and suffer more from vaginal dryness and sexual problems than do those of other ethnicities or people in the USA. Risk of depression is significantly associated with severe climacteric symptoms in women. Despite hormonal treatment being popular among Latin American prescribers, its use is low, and natural therapies are the preferred method to improve quality of life, such as the use of food rich in omega-3-fatty acids, potassium, or calcium. Low adherence might also be related to lower economic status and lack of a medical prescription.

According to WHO, some key points that could improve the understanding of menopause include raising awareness at individual and societal level; advocating for inclusion of diagnosis, treatment, and counselling; inclusion of training on menopause and treatment options; life course approach to health and wellbeing; and ensuring access to proper health information and services. However, from a social perspective, menopause can be viewed differently depending on the culture, considering Latin America is a heterogenous group of different countries, cultures, nationalities, religion beliefs, immigration histories, diet, and so on. However, ageism and misogyny surround Latin American culture, directly impacting women’s health. The fragmentation and segmentation of health systems and inequalities in health are deeply rooted in Latin American countries. An integrative approach is a helpful way to tackle efficiency and improve public health policies across countries.

Barriers to menopause care are very present in our society. Gender, as a social determinant of health, plays a key part in access to health care and availability of therapies. There is still a long way to change the way women are viewed in the society, especially after they start experiencing menopausal symptoms. Funding for research targeting women’s health is a necessity. An individualised approach is needed so women can make informed decisions on the best available treatment, and this is achievable with information, training, and building an inclusive and supportive society. At *The Lancet Regional Health-Americas* we welcome studies targeting new therapies for menopausal symptoms and the health of those who experience menopause in the Americas region.

